# Patient Outcomes with Teaching Versus Nonteaching Healthcare: A Systematic Review

**DOI:** 10.1371/journal.pmed.0030341

**Published:** 2006-09-12

**Authors:** Panagiotis N Papanikolaou, Georgia D Christidi, John P. A Ioannidis

**Affiliations:** 1 Department of Hygiene and Epidemiology, University of Ioannina School of Medicine, Ioannina, Greece; 2 Institute for Clinical Research and Health Policy Studies, Department of Medicine, Tufts University School of Medicine, Boston, Massachusetts, United States of America; Norwegian Directorate for Health and Social Welfare, Norway

## Abstract

**Background:**

Extensive debate exists in the healthcare community over whether outcomes of medical care at teaching hospitals and other healthcare units are better or worse than those at the respective nonteaching ones. Thus, our goal was to systematically evaluate the evidence pertaining to this question.

**Methods and Findings:**

We reviewed all studies that compared teaching versus nonteaching healthcare structures for mortality or any other patient outcome, regardless of health condition. Studies were retrieved from PubMed, contact with experts, and literature cross-referencing. Data were extracted on setting, patients, data sources, author affiliations, definition of compared groups, types of diagnoses considered, adjusting covariates, and estimates of effect for mortality and for each other outcome. Overall, 132 eligible studies were identified, including 93 on mortality and 61 on other eligible outcomes (22 addressed both). Synthesis of the available adjusted estimates on mortality yielded a summary relative risk of 0.96 (95% confidence interval [CI], 0.93–1.00) for teaching versus nonteaching healthcare structures and 1.04 (95% CI, 0.99–1.10) for minor teaching versus nonteaching ones. There was considerable heterogeneity between studies (I^2^ = 72% for the main analysis). Results were similar in studies using clinical and those using administrative databases. No differences were seen in the 14 studies fully adjusting for volume/experience, severity, and comorbidity (relative risk 1.01). Smaller studies did not differ in their results from larger studies. Differences were seen for some diagnoses (e.g., significantly better survival for breast cancer and cerebrovascular accidents in teaching hospitals and significantly better survival from cholecystectomy in nonteaching hospitals), but these were small in magnitude. Other outcomes were diverse, but typically teaching healthcare structures did not do better than nonteaching ones.

**Conclusions:**

The available data are limited by their nonrandomized design, but overall they do not suggest that a healthcare facility's teaching status on its own markedly improves or worsens patient outcomes. Differences for specific diseases cannot be excluded, but are likely to be small.

## Abstract 2

Published data do not suggest that the teaching status of a hospital or other healthcare facility alone influences the outcome of patients treated in that facility.

## Editors' Summary

**Background.:**

When people need medical treatment they may be given it in a “teaching hospital.” This is a place where student doctors and other trainee healthcare workers are receiving part of their education. They help give some of the treatment that patients receive. Teaching hospitals are usually large establishments and in most countries they are regarded as being among the very best hospitals available, with leading physicians and surgeons among the staff. It is usually assumed that patients who are being treated in a teaching hospital are lucky, because they are getting such high-quality healthcare. However, it has sometimes been suggested that, because some of the people involved in their care are still in training, the patients may face higher risks than those who are in nonteaching hospitals.

**Why Was This Study Done?:**

The researchers wanted to find out which patients do best after treatment—those who were treated in teaching hospitals or those who were in nonteaching hospitals. This is a difficult issue to study. The most reliable way of comparing two types of treatment would be to decide at random which treatment each patient should receive. (For more on this see the link below for “randomized controlled trials.”) In practice, it would be difficult to set up a study where the decision on which hospital a patient should go to was made at random. One problem is that, because of the high reputation of teaching hospitals, the patients whose condition is the most serious are often sent there, with other patients going to nonteaching hospitals. It would not be a fair test to compare the “outcome” for the most seriously ill patients with the outcome for those whose condition was less serious.

**What Did the Researchers Do and Find?:**

The researchers conducted a thorough search for studies that had already been done, which met criteria which the researchers had specified in advance. This type of research is called a “systematic review.” They found 132 studies that had compared the outcomes of patients in teaching or nonteaching hospitals. None of these studies was a trial. (They were “observational studies” where researchers had gathered information on what was already taking place, rather than setting up an experiment.) However, in 14 studies, extensive allowances had been made for differences in such factors as the severity of the patients' condition, and whether or not they had more than one type of illness when they were treated. There was a great deal of variability in the results between the studies but, overall, there was no major difference in the effectiveness of treatment provided by the two types of hospital.

**What Do These Findings Mean?:**

There is no evidence to support that it is better to be given treatment in a teaching or a nonteaching hospital. The authors do note that a limitation in their analysis is that it was based on studies that were not randomized controlled trials. They also raise the question that differences might be found if considering specific diseases one by one, rather than putting information on all conditions together. However, they believe that any such difference would be small. Their findings will be useful in the continuing debate on the most effective ways to train doctors, while at the same time providing the best possible care for patients.

**Additional Information.:**

Please access these Web sites via the online version of this summary at http://dx.doi.org/10.1371/journal.pmed.0030341.

- Wikipedia entry on teaching hospitals (note: Wikipedia is a free online encyclopedia that anyone can edit)
- Information on randomized clinical trials from the US National Institutes of Health
- A definition of systematic reviews from the Cochrane Collaboration, an organization which produces systematic reviews

All of the above include links to other Web sites where more detailed information can be found.

## Introduction

A large number of studies, including many in leading medical journals (see Protocol S1 for references for the studies themselves) [1,2] have tried to address whether medical teaching settings obtain better patient outcomes than nonteaching ones; theoretically a teaching environment may also entail unnecessary risks for patients. Superior outcomes in teaching settings have been reported in some studies, but others have claimed the opposite [3].

The pertinent evidence is derived entirely from nonrandomized studies. However, patient populations in different settings may have different case-mixes—for example, academic centres often receive the most difficult cases [4–6]. Teaching versus nonteaching settings may differ also in structure, e.g., availability of technology or volume of patients [7–11]. To avoid confounding, these covariates need to be accounted for. Healthcare structures may also differ in process characteristics, i.e. measures that address the appropriate implementation of healthcare. These are not patient outcomes, but may translate into differential outcomes, e.g., if the right treatment is used more frequently, patients should do better. A comparison of outcomes in teaching versus nonteaching units should adjust for differences in patients and structures. However, adjustment for process would dilute any true outcome differences, since process explains the outcomes. To further complicate matters, data on outcomes and on adjusting covariates may be derived from different administrative or clinical sources [2]. Clinical data are usually more accurate than administrative data that depend on utilization databases.

To address this issue, it is necessary to examine the impact of these issues across different studies and to try to generate a systematic picture of the available evidence on the comparison between teaching and nonteaching healthcare structures. We set to do this by examining the evidence for diverse patient outcomes.

## Methods

### Eligibility

We considered eligible all controlled studies in which any teaching healthcare structures were compared against nonteaching counterparts on any subjective or objective patient outcome. We considered English-language studies regardless of the unit of the healthcare structure (e.g., hospital, service, physician, health system) and regardless of whether teaching status was a primary or secondary analysis.

Teaching hospitals are sometimes further divided into major and minor teaching ones. This distinction is well defined for US hospitals [1,2]. However, to accommodate non-US studies, we allowed contrasts that may be defined with various terms: e.g., the teaching unit(s) may be termed university, academic, medical school-affiliated, resident service, or service with house staff; and the comparator unit(s) may be termed nonuniversity/community, nonacademic, nonaffiliated, or service without residents and students. Overall, we considered all studies in which the compared units differed in teaching status, regardless of the terminology employed. We did not consider studies where compared units differed on whether they had an academic affiliation or not, but were all employed in teaching (e.g., university versus teaching community hospital). Studies of tertiary versus primary/secondary care or specialists versus generalists were not eligible, unless the distinction also coincided with teaching status.

We considered studies regardless of patient outcomes addressed, but excluded length of stay, cost or financial parameters, and process measures from our consideration. We also excluded overlapping articles (retaining only the one with the most complete information), studies with historical literature controls, and studies published before 1970, since these would be largely irrelevant for current healthcare. We excluded nonoriginal articles (editorials, opinion pieces, reviews), and meeting abstracts.

### Searches

We searched PubMed (last search updated June 2005). Given the multifarious nature of eligible studies, we experimented with a variety of different search strategies and compared their yield for selected articles with capture-recapture methods. Our final search was “(university OR academic OR teaching OR faculty OR medical school OR affiliation) AND (community [title word] OR non-academic OR non-academic OR non-teaching OR non-teaching OR affiliation).” Search of the Cochrane Central Registry of Controlled Trials yielded no additional references. We also contacted experts. First we screened titles and abstracts. Articles deemed potentially relevant were screened in the full text. We also perused reviews and the reference lists of identified eligible articles. Two investigators performed the searches. A third investigator settled discrepancies.

### Outcomes

We defined mortality as the primary outcome, since it is the most important and objective outcome although some variability may still be encountered across studies, e.g., the time frame during which mortality is captured. Furthermore, studies addressed mortality more than any other outcome. We set no restriction on eligibility for secondary patient outcomes, and accepted both objective (e.g., morbidity) and subjective (e.g., patient satisfaction) outcomes.

### Data Extraction

For each eligible study, we extracted information on authors, year of publication, type of data (clinical data where information was obtained directly from medical records versus administrative data from utilization databases), reported academic affiliation of corresponding author (yes, no, unclear), time period for the analysed databases, number of hospitals or healthcare structures, number of patients, and definitions of teaching and nonteaching status. Academic affiliation was derived from addresses of health science-related schools, universities, hospitals, or other institutions at which teaching is a key mission; nonacademic addresses included research or nonresearch institutions and hospitals at which teaching is not a key mission.

For all studies that addressed mortality, we recorded whether any adjustment was made for nonprocess covariates. If so, we recorded the adjusting covariates as well as the adjusted estimate of the relative risk (RR) and 95% confidence interval (CI) for the comparison between teaching versus nonteaching healthcare structures. RRs and 95% CIs were derived from the presented data in various forms depending on the model used for analysis (e.g., Cox hazard ratios, odds ratios from logistic regressions, Poisson relative risks, standardized mortality ratios, etc.) and were computed from the presented information when not directly stated by the authors. When both major and minor (other) teaching categories were available, we recorded separate RR and 95% CI for major teaching versus nonteaching and other teaching versus nonteaching comparisons. When multiple adjusted models were available, we preferred the one with the most extensive nonprocess adjustments. Data were recorded separately for different health conditions, whenever such separate information was provided. When only unadjusted estimates were given, these were recorded but not further analysed because of the problems of unadjusted estimates. For each study that provided adjusted estimates we recorded whether adjustments had addressed volume/experience, severity, and comorbidity—beyond simple considerations of age, gender, urgency of a procedure, and (for multiple-diagnosis studies) diagnosis-related group.

For all studies that addressed any other outcome besides mortality, the same considerations applied for recording adjusted estimates for informative comparisons, selecting adjusted models, and presenting data separately according to each outcome and condition of interest. Unadjusted analyses were simply recorded, as above.

Data extraction was performed by two investigators and further checked by a third senior investigator for accuracy. Discrepancies were discussed to reach consensus.

### Data Synthesis

We summarized descriptive characteristics of the studies. We had anticipated that quantitative synthesis of the retrieved information may be precarious given the diversity in study designs, outcomes, measurements, and adjustments. Nevertheless, mortality was a very common, unambiguous outcome and was reasonably amenable to exploratory quantitative synthesis. Given the expected between-study heterogeneity, all formal meta-analyses were performed using random effects calculations [12] using general variance models. Each study was weighted by the inverse of its variance plus the estimated between-study variance. Between-study heterogeneity was estimated with the Q statistic [12] and the I^2^ statistic [13]. The former is a chi square-based test for the statistical significance of heterogeneity, while the latter provides a measure of the extent of heterogeneity and values above 75 suggest very large heterogeneity.

We performed an overall synthesis using all data on mortality across all studies and separate analyses according to subgroups defined by type of data (clinical versus administrative), year of publication (per decade), location of study (US versus other), affiliation of corresponding author (academic, nonacademic, unclear), and whether the compared healthcare structures were named as teaching versus nonteaching or otherwise. We also performed separate analyses for each health condition where at least three RR estimates were available for data synthesis. A sensitivity analysis synthesized only the more rigorously adjusted studies where adjustments had considered volume/experience, severity, and comorbidity (as defined above). Finally, we examined whether observed effect sizes were related with the precision of the estimates [14]. When less-precise studies show more prominent effects than more precise studies, this may reflect bias (including publication bias), but may also hint to study-design differences or other genuine sources of heterogeneity.

Whenever data compared major teaching, minor (other) teaching, and nonteaching status, we used the major teaching versus nonteaching comparison. We also performed a separate analysis that considered all comparisons of minor teaching versus nonteaching status to address specifically whether healthcare structures that perform limited teaching have different mortality rates than nonteaching ones. No studies specifically defined and compared only minor teaching versus nonteaching healthcare.

Finally, for nonmortality outcomes, we simply described the range of estimates across studies. Analyses were performed in SPSS 12.0 (SPSS, Chicago, Illinois, United States).

## Results

### Retrieval of Articles and Eligible Studies

**Figure 1:**
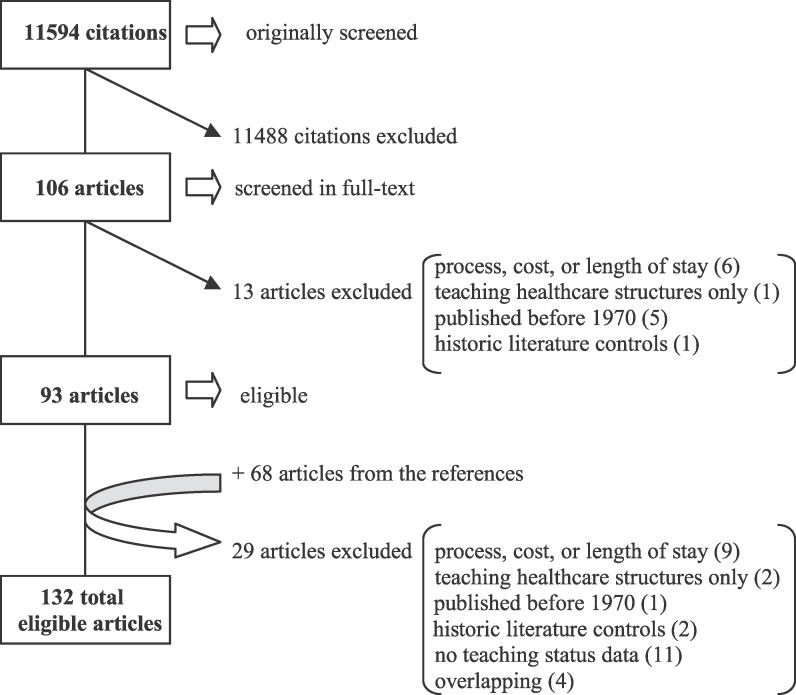
Flow Figure for Screened, Included, and Excluded Articles

Of 11,594 originally screened articles (Figure 1), 132 articles were eventually eligible (Table 1): 93 addressed mortality, and 61 addressed other eligible outcomes (22 addressed both mortality and other outcomes). Most eligible studies were performed in the US (*n* = 94, 71.2%). Studies had also been done in Canada (*n* = 10), European countries (*n* = 19), Australia (*n* = 2), and Asia (*n* = 6), while one study was multinational. Studies covered a wide range of time periods, but 75 (56.8%) studied patient databases after 1991. The word “teaching” was explicitly used in the naming of the compared groups in 97 studies (73.5%), while in the other 35 the teaching versus nonteaching comparison could be indirectly inferred from the definition of the groups.

**Table 1:**
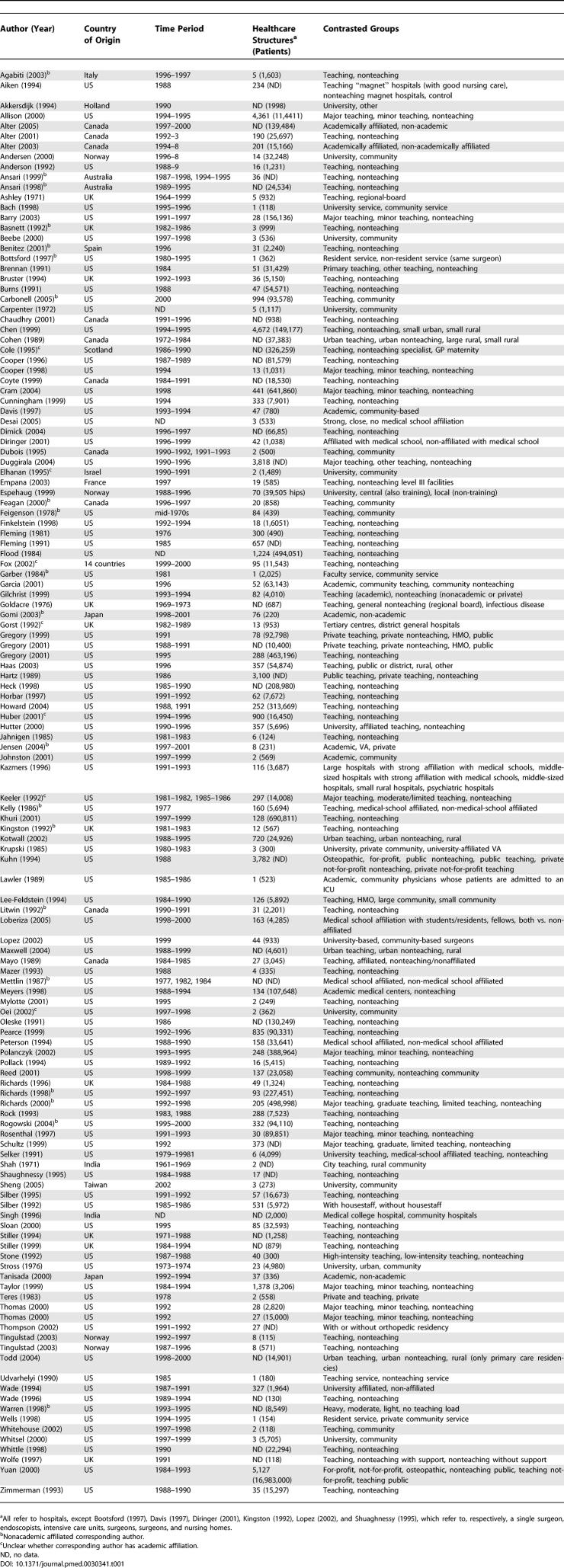
Characteristics of Eligible Studies

Of the 93 articles containing mortality data, 28 (30.1%) provided only unadjusted estimates, and 17 (18.3%) provided adjusted data that were not usable because of lack of sufficient detail in reporting or because the contrast was not directly relevant to the teaching status (e.g., comparison of major teaching versus minor teaching and nonteaching combined). Data on the 48 eligible articles containing adjusted mortality and their adjusting covariates are shown in Table 2. Less than half of them (20 [41.7%]) used clinical data sources. Adjusting covariates varied from 1 to 59 per study. A wide variety of diseases were represented in these studies (Table 2). The 48 articles contained 74 eligible comparisons. Of those comparisons, specific adjustments for volume/experience were performed in 30, specific adjustments for severity were performed in 59, and specific adjustments for comorbidity were performed in 35 comparisons. In 14 comparisons, all three aspects were considered in the adjustments.

**Table 2:**
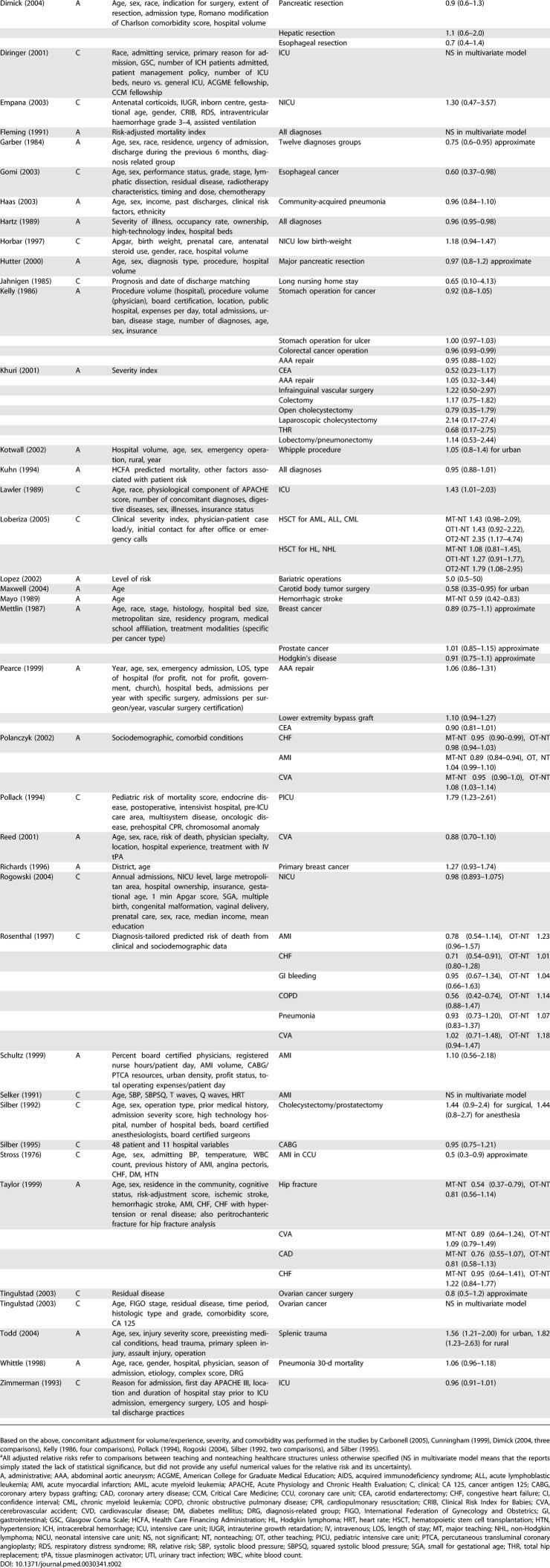
Comparisons of Teaching versus Nonteaching Healthcare on Patient Mortality

Of the 61 studies that addressed other patient outcomes, 23 (37.7%) provided only unadjusted estimates and ten (15.9%) had nonusable adjusted analyses. Adjusted estimates were available for 28 (45.9%) studies (Table 3). Clinical data sources were used in 11 of them (39.3%).

**Table 3:**
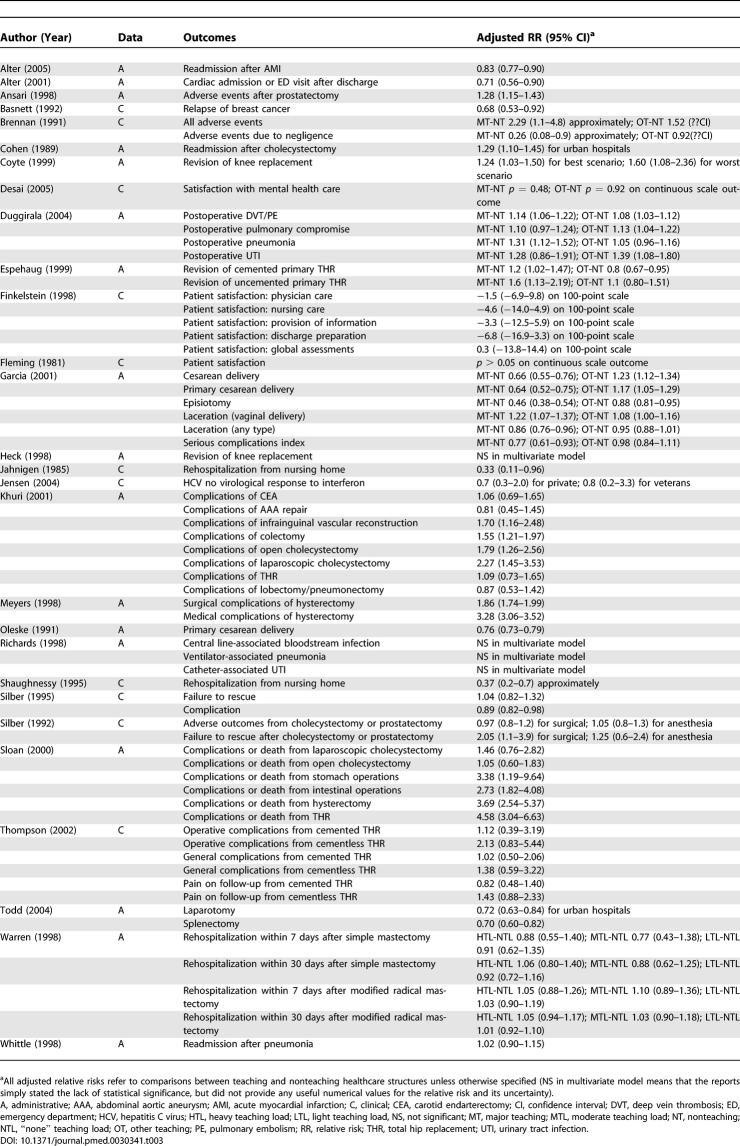
Comparisons of Teaching versus Nonteaching Healthcare on Other Patient Outcomes Besides Mortality

### Mortality

**Figure 2:**
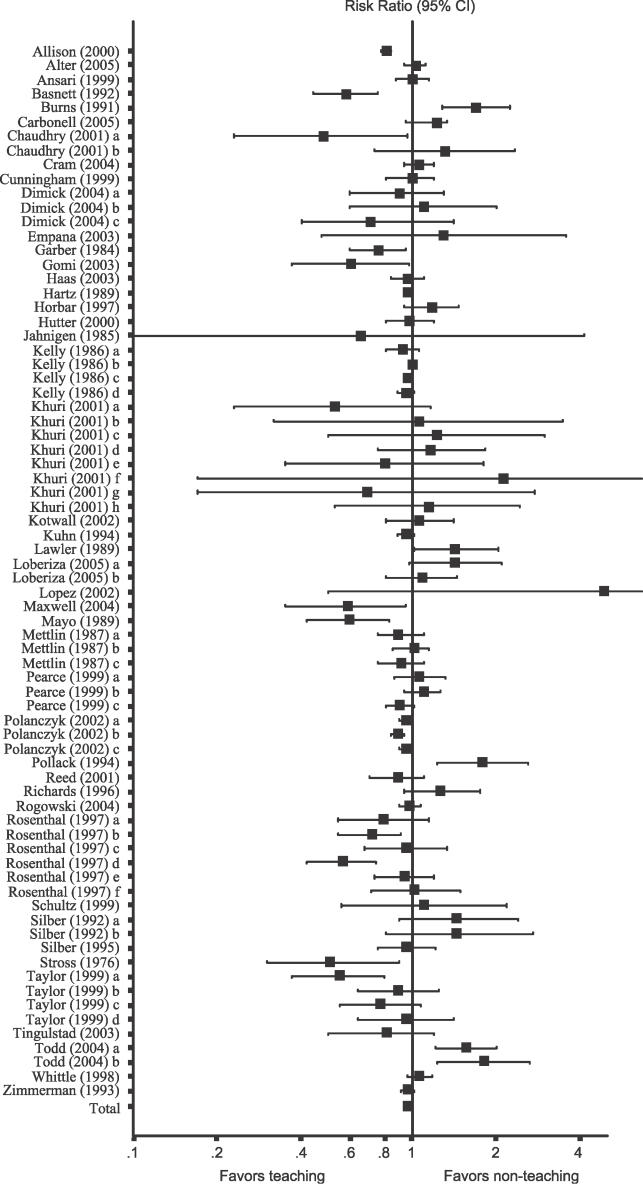
Relative Risk Estimates RR estimates and 95% CIs across studies are shown that address mortality along with summary RR by random effects calculations (Total). The order of the presented estimates is the same as in Table 2. Articles that include estimates on various diagnoses are presented with separate estimates according to type of diagnosis and lettered in the order of Table 2.

Overall synthesis of all usable adjusted mortality data yielded a summary RR of 0.96 (95% CI, 0.93–1.00; *p* = 0.024 [Figure 2]) for teaching versus nonteaching healthcare structures. Between-study heterogeneity was considerable (*p* < 0.001; I^2^ = 72%). However, there was no evidence that studies with smaller weight had different estimates from studies with larger weight (tau correlation coefficient −0.03; *p* = 0.71), and the same was true when administrative database studies were examined separately from clinical database studies. Comparisons between minor teaching healthcare structures and nonteaching ones yielded a RR of 1.04 for mortality (95% CI, 0.99–1.10), also with significant between-study heterogeneity (I^2^ = 60%).

### Subgroup Analyses

With one exception, no evidence indicated that various subgroup estimates differed among themselves (Table 4). The exception was subgroups defined according to year of publication. The single study in the “year of publication” category 1971–80 gave a large benefit in favour of teaching hospitals, while summary effects from subsequent decades indicated no difference between teaching and nonteaching healthcare structures. Results were similar overall in studies using clinical versus administrative data and were not influenced by the affiliation of the corresponding author, the exact naming of the comparison, or the study location, except for a small nonsignificant trend for superior outcomes with teaching institutions in non-US studies. Heterogeneity was sizable within all of these subgroups.

**Table 4:**
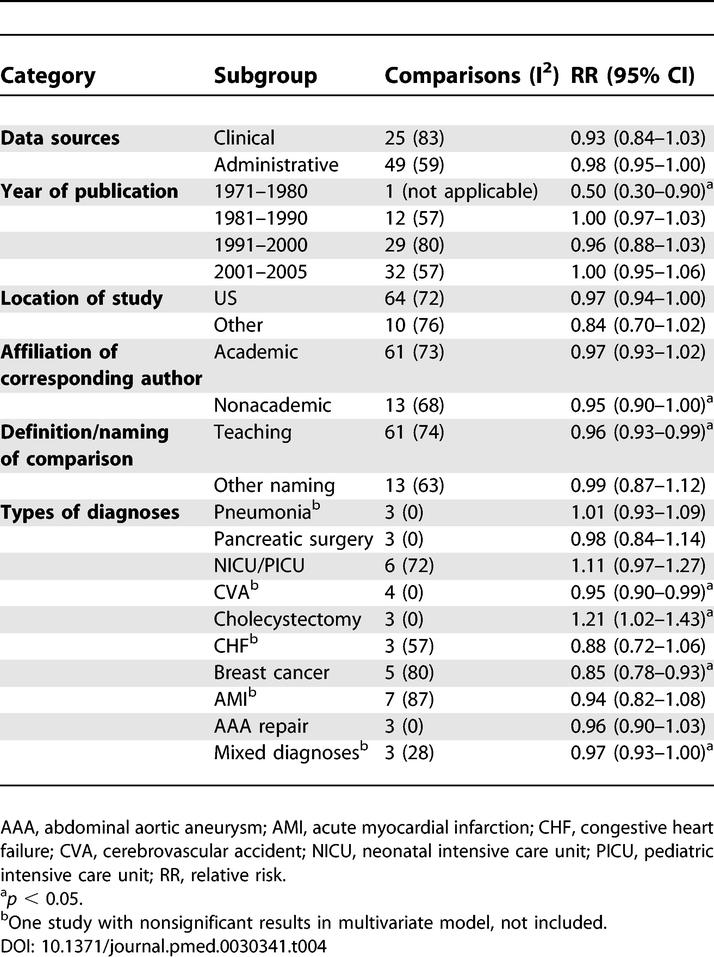
Subgroup Analyses for Mortality

 In diagnosis-focused analyses, there was no between-study heterogeneity for some diagnoses, but heterogeneity persisted for others (Table 4). Certain diagnoses seemed to differ significantly between teaching and nonteaching healthcare structures: Teaching institutions seemed to perform significantly better than nonteaching ones for breast cancer, cerebrovascular accidents, and mixed diagnoses, but for the latter two diagnosis groups the magnitude of the differences was very small. Conversely, a small significant superiority in favour of nonteaching hospitals was seen for cholecystectomy. For most diagnoses, the 95% CIs excluded major differences between teaching and nonteaching healthcare structures.

### Sensitivity Analysis

An analysis limited to the 14 comparisons in which volume/experience, severity, and comorbidity had all been specifically adjusted for yielded a summary RR of 1.01 (95% CI, 0.94–1.07). However, considerable between-study heterogeneity existed (I^2^ = 60%).

Among the 14 comparisons, statistically significant diagnosis-specific differences in mortality were seen in two studies. One study found significantly increased mortality in teaching paediatric intensive care units (RR 1.79; 95% CI, 1.23–2.61). Another study found a small nominally significant reduction in mortality risk in teaching hospitals for colorectal cancer surgery (RR 0.96; 95% CI, 0.93–0.99). None of the conditions for which significant benefits had been seen in teaching healthcare in the overall analysis (cerebrovascular disease, breast cancer, mixed diagnoses) were addressed by any studies with full concomitant adjustment for volume/experience, severity, and comorbidity. Conversely, one study on cholecystectomy outcomes had performed these adjustments and showed similar trends for increased death risk in teaching hospitals (RR 1.21; 95% CI, 0.95–1.34), while another study with two comparisons addressing mortality on cholecystectomy/prostatectomy procedures also found a nonsignificantly increased risk of death in teaching hospitals (RR 1.44 in both comparisons).

### Other Outcomes

Types of outcomes that were addressed in the included studies varied greatly, precluding any formal quantitative synthesis. Overall, among estimates presented with odds ratios, 36 were in favour of nonteaching hospitals and 21 were in favour of teaching hospitals, but most of these estimates were not statistically significant (Table 3). None of the six continuous outcome estimates pertaining to patient satisfaction reached statistical significance.

## Discussion

This systematic review of 132 studies revealed little evidence for a difference in healthcare outcomes between teaching and nonteaching settings. Observational study designs have limitations, but our results do indicate that differences in mortality outcomes between teaching and nonteaching healthcare structures, if they exist, appear small, and may not exist at all for most diseases and circumstances. Summary RR increase or decrease of 4% is well within the range of error expected from observational studies. Focusing on formal statistical significance would be misleading here [15], and the precision of effect sizes should not be overinterpreted. Given the wide diversity of these studies, the quantitative results should only be seen as suggestive, not conclusive. Results on nonmortality outcomes were even more diverse, further limiting quantitative inferences, but nonteaching hospitals did not seem to have inferior performance in most cases. Our results suggest in broad terms that teaching hospital status does not in and of itself result in major benefits nor risks for patient outcomes. In addition to the results of the combined analysis, which should be interpreted with great caution, our systematic review highlights some of the major problems in this literature.

We observed large between-study heterogeneity. This is expected, given the nonrandomized design of these studies and the variability in settings, diseases, adjusting factors, and databases used. Multiple comparisons, selective reporting, publication bias, and other study-specific biases are an additional threat to this literature and it would be difficult to probe their exact depth. We observed some small differences between teaching and nonteaching institutions for certain diagnoses. One may argue that differences in patient outcomes with teaching versus nonteaching healthcare are expected to exist only for specific diseases and settings. Focusing on subgroup analyses, however, can lead to misleading claims when the overall data are unavoidably weak due to inherent design problems.

Allowing for this caveat, better survival was seen for breast cancer and possibly cerebrovascular accidents in teaching healthcare. Unfortunately, these superior outcomes with teaching hospitals were seen in studies that did not adjust for volume/experience, severity, as well as comorbidity. Teaching centres may have better experience and closer adherence to guidelines for treating some types of cancer patients [16] and may utilize treatment more appropriately for some vascular diseases [17]. Conversely, for cholecystectomy the mortality rates were lower in nonteaching healthcare. Involvement of inexperienced trainees may not be beneficial for patients undergoing a common operation, such as cholecystectomy, in which experience is the most important factor. In another study, lack of experience of residents was also felt to underlie the relatively poor outcomes of teaching hospitals in paediatric intensive care patients [18]. However, such differential results should be interpreted very cautiously. It is impossible to adjust for all potential confounders in such studies.

Additional caveats should be discussed. We believe that some eligible studies are still missing from our evaluation, since it is very difficult to identify all articles that have attempted incidentally a cursory comparison of teaching versus nonteaching healthcare. Two relatively recent systematic reviews in this field found fewer than 25 eligible articles each, probably because of this limitation [2,3]. Focusing on studies in which teaching effects are claimed to be primary findings may bias the results in favour of teaching institutions. However, even if we missed some studies in our assessment, the data that we managed to find represent substantial evidence, and conclusions are unlikely to change.

Several studies provided unadjusted estimates of the RRs. Analyzing unadjusted estimates is problematic, since they do not consider differences in case-mix, baseline severity, and other patient characteristics. Thus, we used only adjusted estimates of the RRs for our data synthesis. Even these estimates may be biased. There is no way to correct for all possible confounding in observational designs. Volume and experience with the management of a disease are also important variables to adjust for [7–11,19]. However, even for confounding factors that seem to be very important, their exact impact is not yet fully known, and recent better-quality studies have begun to reveal the inadequacies of previous work [20]. Studies with adjustments for the most important covariates yielded similar results to the overall meta-analysis, but even here residual confounding cannot be fully excluded. Furthermore, the quality of the data may sometimes have been less than optimal, in particular when the data sources were administrative rather than clinical. Nevertheless, we found no major differences in the results of studies using clinical versus administrative data sources.

The results of this study may provide enough evidence to fuel the debate on the prospects of academic medicine [21]. Various scenarios for the future of academic medicine have been proposed, according to which academic medicine may eventually be abolished; may become more driven by public dictates; may become more privatized and corporate; may acquire a more global outlook; or may try to be as fully engaged as possible [21]. For those proposing that academic medicine can be abolished, our systematic review may be interpreted as evidence that abolishing teaching versus nonteaching distinctions likely will not affect patient outcomes on average. If public pressure becomes more important, academic medicine may focus more on neglected outcomes of indigent populations. A privatized academic medicine scenario may cause the outcomes of certain unprofitable procedures to receive little attention, while other, more profitable conditions may receive a disproportionately large amount of attention.

The net outcome effects of any change in direction of academic medicine are not easy to predict. Our review is largely limited to data from developed countries. Thus, for example, proposing that strengthening academic medicine in developing countries will not improve patient outcomes should not be done lightly. Teaching in a healthcare setting does not affect only patients and only in the immediate term; it is an integral part of medicine with benefits to patients in the long term as well. As such, teaching should be fostered to create better practitioners in the future for both academic and nonacademic centres.

## Supporting Information

Protocol S1 References for the 132 Eligible Studies Cited in This Analysis (66 KB DOC)

Table S1 Excluded Studies and Reasons for Exclusion (61 KB DOC)

Table S2 Eligible Studies Containing Only Unadjusted or Nonusable Data on Mortality (41 KB DOC)

## Abbreviations

CI: confidence interval
RR: relative risk
