# Supplementary material for: Patient Outcomes with Teaching Versus Nonteaching Healthcare: A Systematic Review
Source: PLoS Med. 2006 Sep 12;3(9):e341. doi: 10.1371/journal.pmed.0030341 (PMC1564172; doi:10.1371/journal.pmed.0030341)
Supplement: Supplementary file 1 [file pmed.0030341.sd001.doc]

APPENDIX: REFERENCES OF THE 132 ELIGIBLE STUDIES

1. Agabiti N, Ancona C, Forastiere F, Arca M, Perucci CA. Evaluating outcomes of hospital care following coronary artery bypass surgery in Rome, Italy. Eur J Cardiothorac Surg 2003 Apr;23(4):599-606; discussion 607-8.
2. Aiken LH, Smith HL, Lake ET. Lower Medicare mortality among a set of hospitals known for good nursing care. Med Care 1994 Aug;32(8):771-87.
3. Akkersdijk GJ, van der Graaf Y, van Bockel JH, de Vries AC, Eikelboom BC. Mortality rates associated with operative treatment of infrarenal abdominal aortic aneurysm in The Netherlands. Br J Surg 1994 May;81(5):706-9.
4. Allison JJ, Kiefe CI, Weissman NW, Person SD, Rousculp M, Canto JG, Bae S, Williams OD, Farmer R, Centor RM. Relationship of hospital teaching status with quality of care and mortality for Medicare patients with acute MI. JAMA 2000 Sep 13;284(10):1256-62.
5. Alter DA, Austin PC, Tu JV; Canadian Cardiovascular Outcomes Research Team. Community factors, hospital characteristics and inter-regional outcome variations following acute myocardial infarction in Canada. Can J Cardiol 2005 Mar;21(3):247-55.
6. Alter DA, Naylor CD, Austin PC, Tu JV. Long-term MI outcomes at hospitals with or without on-site revascularization. JAMA 2001 Apr 25;285(16):2101-8.
7. Alter DA, Tu JV, Austin PC, Naylor CD. Waiting times, revascularization modality, and outcomes after acute myocardial infarction at hospitals with and without on-site revascularization facilities in Canada. J Am Coll Cardiol 2003 Aug 6;42(3):410-9.
8. Andersen BM, Ringertz SH, Gullord TP, Hermansen W, Lelek M, Norman BI, Nystad MT, Rod KA, Roed RT, Smidesang IJ, Solheim N, Tandberg S, Halsnes R, Wenche Hoystad M. A three-year survey of nosocomial and community-acquired infections, antibiotic treatment and re-hospitalization in a Norwegian health region. J Hosp Infect 2000 Mar;44(3):214-23.
9. Anderson FA Jr, Wheeler HB. Physician practices in the management of venous thromboembolism: a community-wide survey. J Vasc Surg 1992 Nov;16(5):707-14.
10. Ansari MZ, Ackland MJ, Jolley DJ, Carson N, McDonald IG. Inter-hospital comparison of mortality rates. Int J Qual Health Care 1999 Feb;11(1):29-35.
11. Ansari MZ, Costello AJ, Jolley DJ, Ackland MJ, Carson N, McDonald IG. Adverse events after prostatectomy in Victorian public hospitals. Aust N Z J Surg 1998 Dec;68(12):830-6.
12. Ashley JS, Howlett A, Morris JN. Case-fatality of hyperplasia of the prostate in two teaching and three regional-board hospitals. Lancet 1971 Dec 11;2(7737):1308-11.
13. Bach PB, Carson SS, Leff A. Outcomes and resource utilization for patients with prolonged critical illness managed by university-based or community-based subspecialists. Am J Respir Crit Care Med 1998 Nov;158(5 Pt 1):1410-5.
14. Barry WA, Rosenthal GE. Is there a July phenomenon? The effect of July admission on intensive care mortality and length of stay in teaching hospitals. J Gen Intern Med 2003 Aug;18(8):639-45.
15. Basnett I, Gill M, Tobias JS. Variations in breast cancer management between a teaching and a non-teaching district. Eur J Cancer 1992;28A(12):1945-50.
16. Beebe LA, Rayburn WF, Beaty CM, Eberly KL, Stanley JR, Rayburn LA. Indications for labor induction. Differences between university and community hospitals. J Reprod Med 2000 Jun;45(6):469-75.
17. Benitez M, Codina N, Dalfo A, Vila MA, Escriba JM, Senar E, Miracle M, Tovillas FJ. Control of blood pressure in a population of patients with hypertension and in a subgroup with hypertension and diabetes: relationship with characteristics of the health care center and the community. Aten Primaria 2001 Oct 15;28(6):373-80.
18. Bottsford JH, Blake R, Small T, Bottsford J. Carotid endarterectomy: the outcome is not adversely affected by surgical residents. J S C Med Assoc 1997 Jul;93(7):243-7.
19. Brennan TA, Hebert LE, Laird NM, Lawthers A, Thorpe KE, Leape LL, Localio AR, Lipsitz SR, Newhouse JP, Weiler PC, et al. Hospital characteristics associated with adverse events and substandard care. JAMA 1991 Jun 26;265(24):3265-9.
20. Bruster S, Jarman B, Bosanquet N, Weston D, Erens R, Delbanco TL. National survey of hospital patients. BMJ 1994 Dec 10;309(6968):1542-6.
21. Burns LR, Wholey DR. The effects of patient, hospital, and physician characteristics on length of stay and mortality. Med Care 1991 Mar;29(3):251-71.
22. Carbonell AM, Lincourt AE, Kercher KW, Matthews BD, Cobb WS, Sing RF, Heniford BT. Do patient or hospital demographics predict cholecystectomy outcomes? A nationwide study of 93,578 patients. Surg Endosc 2005 May 3; [Epub ahead of print]
23. Carpenter RR, Reed DE. The outcome for patients with cerebrovascular disease in university and community hospitals. Stroke 1972 Nov-Dec;3(6):747-58.
24. Chaudhry R, Goel V, Sawka C. Breast cancer survival by teaching status of the initial treating hospital. CMAJ 2001 Jan 23;164(2):183-8. Erratum in: CMAJ 2001 May 1;164(9):1280.
25. Chen J, Radford MJ, Wang Y, Marciniak TA, Krumholz HM. Performance of the '100 top hospitals': what does the report card report? Health Aff (Millwood) 1999 Jul-Aug;18(4):53-68.
26. Cohen MM, Young TK, Hammarstrand KM. Ethnic variation in cholecystectomy rates and outcomes, Manitoba, Canada, 1972-84. Am J Public Health 1989 Jun;79(6):751-5.
27. Cole SK, Macfarlane A. Safety and place of birth in Scotland. J Public Health Med 1995 Mar;17(1):17-24.
28. Cooper GS, Yuan Z, Landefeld CS, Rimm AA. Surgery for colorectal cancer: Race-related differences in rates and survival among Medicare beneficiaries. Am J Public Health 1996 Apr;86(4):582-6.
29. Cooper GS, Chak A, Way LE, Hammar PJ, Harper DL, Rosenthal GE. Endoscopic practice for upper gastrointestinal hemorrhage: differences between major teaching and community-based hospitals. Gastrointest Endosc 1998 Oct;48(4):348-53.
30. Coyte PC, Hawker G, Croxford R, Wright JG. Rates of revision knee replacement in Ontario, Canada. J Bone Joint Surg Am 1999 Jun;81(6):773-82.
31. Cram P, Hillis SL, Barnett M, Rosenthal GE. Effects of weekend admission and hospital teaching status on in-hospital mortality. Am J Med 2004 Aug 1;117(3):151-7.
32. Cunningham WE, Tisnado DM, Lui HH, Nakazono TT, Carlisle DM. The effect of hospital experience on mortality among patients hospitalized with acquired immunodeficiency syndrome in California. Am J Med 1999 Aug;107(2):137-43.
33. Davis WZ, Cotton PB, Arias R, Williams D, Onken JE. ERCP and sphincterotomy in the context of laparoscopic cholecystectomy: academic and community practice patterns and results. Am J Gastroenterol 1997 Apr;92(4):597-601.
34. Desai RA, Dausey DJ, Sernyak M, Rosenheck RA. The effects of federal versus state funding and academic affiliation on mental health services. Adm Policy Ment Health 2005 Jan;32(3):267-83.
35. Dimick JB, Cowan JA Jr, Colletti LM, Upchurch GR Jr. Hospital teaching status and outcomes of complex surgical procedures in the United States. Arch Surg 2004 Feb;139(2):137-41.
36. Diringer MN, Edwards DF. Admission to a neurologic/neurosurgical intensive care unit is associated with reduced mortality rate after intracerebral hemorrhage. Crit Care Med. 2001 Mar;29(3):635-40.
37. Dubois B, Nagy AG, Anderson D, Simpson WT, Appleby JP. Comparison of initial laparoscopic cholecystectomy at a community hospital versus a teaching hospital. Can J Surg 1995 Oct;38(5):439-44.
38. Duggirala AV, Chen FM, Gergen PJ. Postoperative adverse events in teaching and nonteaching hospitals. Fam Med 2004 Jul-Aug;36(7):508-13.
39. Elhanan G, Raz R, Pitlik SD, Sharir R, Konisberger H, Samra Z, Kennes Y, Drucker M, Leibovici L. Bacteraemia in a community and a university hospital. J Antimicrob Chemother 1995 Oct;36(4):681-95.
40. Empana JP, Subtil D, Truffert P. In-hospital mortality of newborn infants born before 33 weeks of gestation depends on the initial level of neonatal care: the EPIPAGE study. Acta Paediatr 2003;92(3):346-51.
41. Espehaug B, Havelin LI, Engesaeter LB, Vollset SE. The effect of hospital-type and operating volume on the survival of hip replacements. A review of 39,505 primary total hip replacements reported to the Norwegian Arthroplasty Register, 1988-1996. Acta Orthop Scand 1999 Feb;70(1):12-8.
42. Feagan BG, Marrie TJ, Lau CY, Wheeler SL, Wong CJ, Vandervoort MK. Treatment and outcomes of community-acquired pneumonia at Canadian hospitals. CMAJ 2000 May 16;162(10):1415-20.
43. Feigenson JS, Feigenson WD, Gitlow HS, McCarthy ML, Greenberg SD. Outcome and cost for stroke patients in academic and community hospitals. Comparison of two groups referred to a regional rehabilitation center. JAMA 1978 Oct 20;240(17):1878-80.
44. Finkelstein BS, Singh J, Silvers JB, Neuhauser D, Rosenthal GE. Patient and hospital characteristics associated with patient assessments of hospital obstetrical care. Med Care 1998 Aug;36(8 Suppl):AS68-78.
45. Fleming GV. Hospital structure and consumer satisfaction. Health Serv Res 1981;16(1):43-63.
46. Fleming ST, McMahon LF Jr, Desharnais SI, Chesney JD, Wroblewski RT. The measurement of mortality. A risk-adjusted variable time window approach. Med Care. 1991 Sep;29(9):815-28.
47. Flood AB, Scott WR, Ewy W. Does practice make perfect? Part II: The relation between volume and outcomes and other hospital characteristics. Med Care 1984 Feb;22(2):115-25.
48. Fox KA, Goodman SG, Klein W, Brieger D, Steg PG, Dabbous O, Avezum A. Management of acute coronary syndromes. Variations in practice and outcome; findings from the Global Registry of Acute Coronary Events (GRACE). Eur Heart J 2002 Aug;23(15):1177-89.
49. Garber AM, Fuchs VR, Silverman JF. Case mix, costs, and outcomes. Differences between faculty and community services in a university hospital. N Engl J Med 1984 May 10;310(19):1231-7.
50. Garcia FA, Miller HB, Huggins GR, Gordon TA. Effect of academic affiliation and obstetric volume on clinical outcome and cost of childbirth. Obstet Gynecol 2001 Apr;97(4):567-76.
51. Gilchrist IC, Gardner LH, Muhlestein JB, Arnold AM, Lincoff AM, Califf RM, Tcheng JE, Topol EJ. Effect of institutional volume and academic status on outcomes of coronary interventions: the IMPACT-II experience. Am Heart J 1999 Nov;138(5 Pt 1):976-82.
52. Goldacre MJ. Acute bacterial meningitis: where do children die? Int J Epidemiol 1976 Dec;5(4):343-7.
53. Gomi K, Oguchi M, Hirokawa Y, Kenjo M, Ogata T, Takahashi Y, Nakamura N, Yamashita T, Teshima T, Inoue T; Japanese Patterns of Care Study Working Subgroup of Esophageal Cancer. Process and preliminary outcome of a patterns-of-care study of esophageal cancer in Japan: patients treated with surgery and radiotherapy. Int J Radiat Oncol Biol Phys 2003 Jul 1;56(3):813-22.
54. Gorst DW, Johnson KW. Survival in adult leukaemia. Clin Lab Haematol 1992;14(2):99-108.
55. Gregory KD, Ramicone E, Chan L, Kahn KL. Cesarean deliveries for medicaid patients: a comparison in public and private hospitals in Los Angeles county. Am J Obstet Gynecol 1999 May;180(5):1177-84.
56. Gregory KD, Korst LM, Krychman M, Cane P, Platt LD. Variation in vaginal breech delivery rates by hospital type. Obstet Gynecol 2001 Mar;97(3):385-90.
57. Gregory KD, Korst LM, Platt LD. Variation in elective primary cesarean delivery by patient and hospital factors. Am J Obstet Gynecol 2001 Jun;184(7):1521-32; discussion 1532-4.
58. Haas JS, Dean ML, Hung Y, Rennie DJ. Differences in mortality among patients with community-acquired pneumonia in California by ethnicity and hospital characteristics. Am J Med 2003 Jun 1;114(8):660-4.
59. Hartz AJ, Krakauer H, Kuhn EM, Young M, Jacobsen SJ, Gay G, Muenz L, Katzoff M, Bailey RC, Rimm AA. Hospital characteristics and mortality rates. N Engl J Med 1989 Dec 21;321(25):1720-5.
60. Heck DA, Melfi CA, Mamlin LA, Katz BP, Arthur DS, Dittus RS, Freund DA. Revision rates after knee replacement in the United States. Med Care 1998 May;36(5):661-9.
61. Horbar JD, Badger GJ, Lewit EM, Rogowski J, Shiono PH. Hospital and patient characteristics associated with variation in 28-day mortality rates for very low birth weight infants. Vermont Oxford Network. Pediatrics 1997 Feb;99(2):149-56.
62. Howard DL, Silber JH, Jobes DR. Do regulations limiting residents' work hours affect patient mortality? J Gen Intern Med 2004 Jan;19(1):1-7. Erratum in: J Gen Intern Med 2004 Feb;19(2):204. Silber, Jeffery H [corrected to Silber, Jeffrey H].
63. Huber TS, Wang JG, Derrow AE, Dame DA, Ozaki CK, Zelenock GB, Flynn TC, Seeger JM. Experience in the United States with intact abdominal aortic aneurysm repair. J Vasc Surg 2001 Feb;33(2):304-10
64. Hutter MM, Glasgow RE, Mulvihill SJ. Does the participation of a surgical trainee adversely impact patient outcomes? A study of major pancreatic resections in California. Surgery 2000 Aug;128(2):286-92.
65. Jahnigen DW, Kramer AM, Robbins LJ, Klingbeil H, DeVore P. Academic affiliation with a nursing home. Impact on patient outcome. J Am Geriatr Soc 1985 Jul;33(7):472-8.
66. Jensen DM, Cotler SJ, Lam H, Harb G, Shillington A. A comparison of hepatitis C treatment and outcomes at academic, private and Veterans' Affairs treatment centres. Aliment Pharmacol Ther 2004 Jan 1;19(1):69-77.
67. Johnston DC, Chapman KM, Goldstein LB. Low rate of complications of cerebral angiography in routine clinical practice. Neurology 2001 Dec 11;57(11):2012-4.
68. Kazmers A, Jacobs L, Perkins A, Lindenauer SM, Bates E. Abdominal aortic aneurysm repair in Veterans Affairs medical centers. J Vasc Surg 1996 Feb;23(2):191-200.
69. Keeler EB, Rubenstein LV, Kahn KL, Draper D, Harrison ER, McGinty MJ, Rogers WH, Brook RH. Hospital characteristics and quality of care. JAMA 1992 Oct 7;268(13):1709-14.
70. Kelly JV, Hellinger FJ. Physician and hospital factors associated with mortality of surgical patients. Med Care 1986 Sep;24(9):785-800.
71. Khuri SF, Najjar SF, Daley J, Krasnicka B, Hossain M, Henderson WG, Aust JB, Bass B, Bishop MJ, Demakis J, DePalma R, Fabri PJ, Fink A, Gibbs J, Grover F, Hammermeister K, McDonald G, Neumayer L, Roswell RH, Spencer J, Turnage RH; VA National Surgical Quality Improvement Program. Comparison of surgical outcomes between teaching and nonteaching hospitals in the Department of Veterans Affairs. Ann Surg 2001 Sep;234(3):370-82; discussion 382-3.
72. Kingston RD, Walsh S, Jeacock J. Colorectal surgeons in district general hospitals produce similar survival outcomes to their teaching hospital colleagues: review of 5-year survivals in Manchester. J R Coll Surg Edinb 1992 Aug;37(4):235-7.
73. Kotwall CA, Maxwell JG, Brinker CC, Koch GG, Covington DL. National estimates of mortality rates for radical pancreaticoduodenectomy in 25,000 patients. Ann Surg Oncol 2002 Nov;9(9):847-54.
74. Krupski WC, Effeney DJ, Goldstone J, Deuel M, Webb RL, Etheredge SN, Ehrenfeld WK, Stoney RJ. Carotid endarterectomy in a metropolitan community: comparison of results from three institutions. Surgery 1985 Sep;98(3):492-9.
75. Kuhn EM, Hartz AJ, Krakauer H, Bailey RC, Rimm AA. The relationship of hospital ownership and teaching status to 30- and 180-day adjusted mortality rates. Med Care 1994 Nov;32(11):1098-108.
76. Lawler FH, Horner RD, Hainer BL. Process and outcome of critical care provided by community and academic primary care physicians. Fam Med 1989 Jul-Aug;21(4):268-72.
77. Lee-Feldstein A, Anton-Culver H, Feldstein PJ. Treatment differences and other prognostic factors related to breast cancer survival. Delivery systems and medical outcomes. JAMA 1994 Apr 20;271(15):1163-8.
78. Litwin DE, Girotti MJ, Poulin EC, Mamazza J, Nagy AG. Laparoscopic cholecystectomy: trans-Canada experience with 2201 cases. Can J Surg 1992 Jun;35(3):291-6.
79. Loberiza FR Jr, Zhang MJ, Lee SJ, Klein JP, LeMaistre CF, Serna DS, Eapen M, Bredeson CN, Horowitz MM, Rizzo JD. Association of transplant center and physician factors on mortality after hematopoietic stem cell transplantation in the United States. Blood 2005 Apr 1;105(7):2979-87. Epub 2004 Dec 14.
80. Lopez J, Sung J, Anderson W, Stone J, Gallagher S, Shapiro D, Rosemurgy A, Murr MM. Is bariatric surgery safe in academic centers? Am Surg 2002 Sep;68(9):820-3.
81. Maxwell JG, Jones SW, Wilson E, Kotwall CA, Hall T, Hamann S, Brinker CC. Carotid body tumor excisions: adverse outcomes of adding carotid endarterectomy. J Am Coll Surg 2004 Jan;198(1):36-41.
82. Mayo NE, Hendlisz J, Goldberg MS, Korner-Bitensky N, Becker R, Coopersmith H. Destinations of stroke patients discharged from the Montreal area acute-care hospitals. Stroke 1989 Mar;20(3):351-6.
83. Mazer CD, Byrick RJ, Sibbald WJ, Chovaz PM, Goodman SJ, Girotti MJ, Hall JK, Pagliarello J. Postoperative utilization of critical care services by cardiac surgery: a multicenter study in the Canadian healthcare system. Crit Care Med 1993 Jun;21(6):851-9.
84. Mettlin CJ, Schoenfeld ER, Natarajan N, Suh O. Interhospital differences in cancer survival. J Chronic Dis 1987;40(2):157-64.
85. Meyers ER, Steege JF. Risk adjustment for complications of hysterectomy: utility of routinely collected administrative data. Prim Care Update Ob Gyns 1998 Jul 1;5(4):202-203.
86. Mylotte JM, Kahler L, McCann C. Community-acquired bacteremia at a teaching versus a nonteaching hospital: impact of acute severity of illness on 30-day mortality. Am J Infect Control 2001 Feb;29(1):13-9.
87. Oei TT, Dulai GS, Gralnek IM, Chang D, Kilbourne AM, Sale GA. Hospital care for low-risk patients with acute, nonvariceal upper GI hemorrhage: a comparison of neighboring community and tertiary care centers. Am J Gastroenterol 2002 Sep;97(9):2271-8.
88. Oleske DM, Glandon GL, Giacomelli GJ, Hohmann SF. The cesarean birth rate: influence of hospital teaching status. Health Serv Res 1991 Aug;26(3):325-37.
89. Pearce WH, Parker MA, Feinglass J, Ujiki M, Manheim LM. The importance of surgeon volume and training in outcomes for vascular surgical procedures. J Vasc Surg 1999 May;29(5):768-76; discussion 777-8.
90. Peterson ED, Wright SM, Daley J, Thibault GE. Racial variation in cardiac procedure use and survival following acute myocardial infarction in the Department of Veterans Affairs. JAMA 1994 Apr 20;271(15):1175-80.
91. Polanczyk CA, Lane A, Coburn M, Philbin EF, Dec GW, DiSalvo TG. Hospital outcomes in major teaching, minor teaching, and nonteaching hospitals in New York state. Am J Med 2002 Mar;112(4):255-61.
92. Pollack MM, Cuerdon TT, Patel KM, Ruttimann UE, Getson PR, Levetown M. Impact of quality-of-care factors on pediatric intensive care unit mortality. JAMA 1994 Sep 28;272(12):941-6.
93. Reed SD, Cramer SC, Blough DK, Meyer K, Jarvik JG. Treatment with tissue plasminogen activator and inpatient mortality rates for patients with ischemic stroke treated in community hospitals. Stroke 2001 Aug;32(8):1832-40.
94. Richards MA, Wolfe CD, Tilling K, Barton J, Bourne HM, Gregory WM. Variations in the management and survival of women under 50 years with breast cancer in the South East Thames region. Br J Cancer 1996 Mar;73(6):751-7.
95. Richards MJ, Edwards JR, Culver DH, Gaynes RP. Nosocomial infections in coronary care units in the United States. National Nosocomial Infections Surveillance System. Am J Cardiol 1998 Sep 15;82(6):789-93.
96. Richards MJ, Edwards JR, Culver DH, Gaynes RP. Nosocomial infections in combined medical-surgical intensive care units in the United States. Infect Control Hosp Epidemiol 2000 Aug;21(8):510-5.
97. Rock SM. Variability and consistency of rates of primary and repeat cesarean sections among hospitals in two states. Public Health Rep 1993 Jul-Aug;108(4):514-6.
98. Rogowski JA, Horbar JD, Staiger DO, Kenny M, Carpenter J, Geppert J. Indirect vs direct hospital quality indicators for very low-birth-weight infants. JAMA 2004 Jan 14;291(2):202-9.
99. Rosenthal GE, Harper DL, Quinn LM, Cooper GS. Severity-adjusted mortality and length of stay in teaching and nonteaching hospitals. Results of a regional study. JAMA 1997 Aug 13;278(6):485-90.
100. Schultz MA, van Servellen G, Litwin MS, McLaughlin EJ, Uman GC. Can hospital structural and financial characteristics explain variations in mortality caused by acute myocardial infarction? Appl Nurs Res 1999 Nov;12(4):210-4.
101. Selker HP, Griffith JL, D'Agostino RB. A time-insensitive predictive instrument for acute myocardial infarction mortality: a multicenter study. Med Care 1991 Dec;29(12):1196-211.
102. Shah KP, de Souza JM, Sawardeker D, Aphale RV. Comparative study of maternal mortality in rural community and city teaching institution. Indian J Public Health 1971 Jul;15(3):83-91.
103. Shaughnessy PW, Kramer AM, Hittle DF, Steiner JF. Quality of care in teaching nursing homes: findings and implications. Health Care Financ Rev 1995;16(4):55-83.
104. Sheng WH, Wang JT, Lu DC, Chie WC, Chen YC, Chang SC. Comparative impact of hospital-acquired infections on medical costs, length of hospital stay and outcome between community hospitals and medical centres. J Hosp Infect 2005 Mar;59(3):205-14.
105. Silber JH, Rosenbaum PR, Schwartz JS, Ross RN, Williams SV. Evaluation of the complication rate as a measure of quality of care in coronary artery bypass graft surgery. JAMA 1995 Jul 26;274(4):317-23.
106. Silber JH, Williams SV, Krakauer H, Schwartz JS. Hospital and patient characteristics associated with death after surgery. A study of adverse occurrence and failure to rescue. Med Care 1992 Jul;30(7):615-29.
107. Singh UK, Srivastava SP, Kumar A, Thakur AK, Prasad R, Chakrabarti B. Comparative study of perinatal mortality and morbidity in the community and at Medical College Hospital, Patna. Indian Pediatr 1996 Dec;33(12):1057-8.
108. Sloan FA, Conover CJ, Provenzale D. Hospital credentialing and quality of care. Soc Sci Med 2000 Jan;50(1):77-88.
109. Stiller CA, Eatock EM. Survival from acute non-lymphocytic leukaemia, 1971-88: a population based study. Arch Dis Child 1994 Mar;70(3):219-23.
110. Stiller CA, Benjamin S, Cartwright RA, Clough JV, Gorst DW, Kroll ME, Ross JR, Wheatley K, Whittaker JA, Taylor PR, Proctor SJ. Patterns of care and survival for adolescents and young adults with acute leukaemia--a population-based study. Br J Cancer 1999 Feb;79(3-4):658-65.
111. Stone VE, Seage GR 3rd, Hertz T, Epstein AM. The relation between hospital experience and mortality for patients with AIDS. JAMA 1992 Nov 18;268(19):2655-61.
112. Stross JK, Willis PW 3rd, Reynolds EW Jr, Lewis RE, Schatz IJ, Bellfy LC, Copp J. Effectiveness of coronary care units in small community hospitals. Ann Intern Med 1976 Dec;85(6):709-13.
113. Tanisada K, Teshima T, Ikeda H, Abe M, Owen JB, Hanks GE, Yamashita T, Nishio M, Yamada S, Sakai K, Hiraoka M, Hirokawa Y, Oguchi M, Inoue T. A preliminary outcome analysis of the Patterns of Care Study in Japan for esophageal cancer patients with special reference to age: non surgery group. Int J Radiat Oncol Biol Phys 2000 Mar 15;46(5):1223-33.
114. Taylor DH Jr, Whellan DJ, Sloan FA. Effects of admission to a teaching hospital on the cost and quality of care for Medicare beneficiaries. N Engl J Med 1999 Jan 28;340(4):293-9.
115. Teres D, Brown RB, Lemeshow S, Parsells JL. A comparison of mortality and charges in two differently staffed intensive care units. Inquiry 1983;20(3):282-9.
116. Thomas EJ, Orav EJ, Brennan TA. Hospital ownership and preventable adverse events. J Gen Intern Med 2000 Apr;15(4):211-9.
117. Thomas EJ, Orav EJ, Brennan TA. Hospital ownership and preventable adverse events. Int J Health Serv 2000;30(4):745-61.
118. Thompson R, Kane RL, Gromala T, McLaughlin B, Flood S, Morris N, Borbas C. Complications and short-term outcomes associated with total hip arthroplasty in teaching and community hospitals. J Arthroplasty 2002 Jan;17(1):32-40.
119. Tingulstad S, Skjeldestad FE, Hagen B. The effect of centralization of primary surgery on survival in ovarian cancer patients. Obstet Gynecol 2003 Sep;102(3):499-505.
120. Tingulstad S, Skjeldestad FE, Halvorsen TB, Hagen B. Survival and prognostic factors in patients with ovarian cancer. Obstet Gynecol 2003 May;101(5 Pt 1):885-91.
121. Todd SR, Arthur M, Newgard C, Hedges JR, Mullins RJ. Hospital factors associated with splenectomy for splenic injury: a national perspective. J Trauma 2004 Nov;57(5):1065-71.
122. Udvarhelyi IS, Rosborough T, Lofgren RP, Lurie N, Epstein AM. Teaching status and resource use for patients with acute myocardial infarction: a new look at the indirect costs of graduate medical education. Am J Public Health 1990 Sep;80(9):1095-100.
123. Wade TP, Radford DM, Virgo KS, Johnson FE. Complications and outcomes in the treatment of pancreatic adenocarcinoma in the United States veteran. J Am Coll Surg 1994 Jul;179(1):38-48.
124. Wade TP, Halaby IA, Stapleton DR, Virgo KS, Johnson FE. Population-based analysis of treatment of pancreatic cancer and Whipple resection: Department of Defense hospitals, 1989-1994. Surgery 1996 Oct;120(4):680-5; discussion 686-7.
125. Warren JL, Riley GF, Potosky AL, Klabunde CN, Richter E, Ballard-Barbash R. Trends and outcomes of outpatient mastectomy in elderly women. J Natl Cancer Inst 1998 Jun 3;90(11):833-40.
126. Wells RD, Dahl B, Nilson B. Comparison of the levels of quality of inpatient care delivered by pediatrics residents and by private, community pediatricians at one hospital. Acad Med 1998 Feb;73(2):192-7.
127. Whitehouse JD, Friedman ND, Kirkland KB, Richardson WJ, Sexton DJ. The impact of surgical-site infections following orthopedic surgery at a community hospital and a university hospital: adverse quality of life, excess length of stay, and extra cost. Infect Control Hosp Epidemiol 2002 Apr;23(4):183-9.
128. Whitsel AI, Capeless EC, Abel DE, Stuart GS. Adjustment for case mix in comparisons of cesarean delivery rates: university versus community hospitals in Vermont. Am J Obstet Gynecol 2000 Nov;183(5):1170-5.
129. Whittle J, Lin CJ, Lave JR, Fine MJ, Delaney KM, Joyce DZ, Young WW, Kapoor WN. Relationship of provider characteristics to outcomes, process, and costs of care for community-acquired pneumonia. Med Care 1998 Jul;36(7):977-87.
130. Wolfe CD, Tilling K, Raju KS. Management and survival of ovarian cancer patients in south east England. Eur J Cancer 1997 Oct;33(11):1835-40.
131. Yuan Z, Cooper GS, Einstadter D, Cebul RD, Rimm AA. The association between hospital type and mortality and length of stay: a study of 16.9 million hospitalized Medicare beneficiaries. Med Care 2000 Feb;38(2):231-45.
132. Zimmerman JE, Shortell SM, Knaus WA, Rousseau DM, Wagner DP, Gillies RR, Draper EA, Devers K. Value and cost of teaching hospitals: a prospective, multicenter, inception cohort study. Crit Care Med 1993 Oct;21(10):1432-42.
