# Supplementary material for: Patient Outcomes with Teaching Versus Nonteaching Healthcare: A Systematic Review
Source: PLoS Med. 2006 Sep 12;3(9):e341. doi: 10.1371/journal.pmed.0030341 (PMC1564172; doi:10.1371/journal.pmed.0030341)
Supplement: Supplementary file 2 [file pmed.0030341.st001.doc]

Supplementary information 1: Excluded studies and reasons for exclusion

| **Process, cost, length of stay** | **Overlapping** | **All teaching** | **No teaching status data** | **Published before 1970** | **Literature controls only** |
| --- | --- | --- | --- | --- | --- |
| Ayanian (1998) | Cooper (1996) | McCormick (1999) | Ghali (1998) | Lee (1960) | Horsley (1977) |
| Keenan (1998) | Kahn (1994) | Herbst (1993) | Lave (1996) | Lee (1957) | Cohn (2002) |
| Petrik (2003) | Chen (1999) | Knaus (1986) | Bonett (1991) | Cunningham (1957) | Gaylis (1998) |
| Poses (1998) | Barnett (2002) |  | Madison (2004) | Detweiler (1965) |  |
| Taxis (2003) |  |  | Mehta (2002) | Lamkee (1962) |  |
| Wyatt (1997) |  |  | Stranjalis (1996) | Lipworth (1963) |  |
| Venturini (1999) |  |  | Pollack (1991) |  |  |
| Mathoulin-Pelissier (2000) |  |  | Stremple (1993) |  |  |
| Alter (2003) |  |  | Mangus (1999) |  |  |
| Cox (1994) |  |  | Shortell (1988) |  |  |
| Hamburger (1982) |  |  | Capewell (1996) |  |  |
| Srivastava (2003) |  |  |  |  |  |
| Meurer (1998) |  |  |  |  |  |
| Levin (2000) |  |  |  |  |  |
| Goel (1997) |  |  |  |  |  |

## References of excluded studies

1. Mehta RH, Criger DA, Granger CB, Pieper KK, Califf RM, Topol EJ, Bates ER. Patient outcomes after fibrinolytic therapy for acute myocardial infarction at hospitals with and without coronary revascularization capability. J Am Coll Cardiol 2002 Sep 18;40(6):1034-40; discussion 1041-3.
2. Madison K. Hospital-physician affiliations and patient treatments, expenditures, and outcomes. Health Serv Res 2004 Apr;39(2):257-78.
3. Cohn JH, El-Galley R. Radical prostatectomy in a community practice. J Urol 2002 Jan;167(1):224-8.
4. Levin R, Moy E, Griner PF. Trends in specialized surgical procedures at teaching and nonteaching hospitals. Health Aff (Millwood) 2000 Jan-Feb;19(1):230-8.
5. Srivastava R, Homer CJ. Length of stay for common pediatric conditions: teaching versus nonteaching hospitals. Pediatrics 2003 Aug;112(2):278-81.
6. Gaylis FD, Friedel WE, Armas OA. Radical retropubic prostatectomy outcomes at a community hospital. J Urol 1998 Jan;159(1):167-71. Review. Erratum in: J Urol 1998 Mar;159(3):996.
7. Stranjalis G. Ruptured cerebral aneurysm: influence of specialist and trainee-performed operations on outcome. Acta Neurochir (Wien) 1996;138(9):1067-9.
8. Pollack MM, Alexander SR, Clarke N, Ruttimann UE, Tesselaar HM, Bachulis AC. Improved outcomes from tertiary center pediatric intensive care: a statewide comparison of tertiary and nontertiary care facilities. Crit Care Med 1991 Feb;19(2):150-9.
9. Stremple JF, Bross DS, Davis CL, McDonald GO. Comparison of postoperative mortality in VA and private hospitals. Ann Surg 1993 Mar;217(3):277-85.
10. Mangus RS, Mann NC, Worrall W, Mullins RJ. Statewide variation in the treatment of patients hospitalized with spleen injury. Arch Surg 1999 Dec;134(12):1378-84.
11. Shortell SM, Hughes EF. The effects of regulation, competition, and ownership on mortality rates among hospital inpatients. N Engl J Med 1988 Apr 28;318(17):1100-7.
12. Capewell S, Kendrick S, Boyd J, Cohen G, Juszczak E, Clarke J. Measuring outcomes: one month survival after acute myocardial infarction in Scotland. Heart 1996 Jul;76(1):70-5.
13. Lamkee MJ, Donaldson LB, De Alvarez RR. Analysis of cesarean sections in a teaching and a nonteaching hospital. Am J Obstet Gynecol 1962 Mar 1;83:619-31.
14. Detweiler JA. Thyroiditis: Private practice compared with community hospital and university hospital. JAMA 1965 Jun 21;192:1098-100.
15. Cunningham AA, Leask LR. Fatality from two common surgical conditions in teaching and non-teaching hospitals. Lancet 1957 Dec 28;273(7009):1336-7.
16. Lee JA, Morrison SL, Morris JN. Fatality from three common surgical conditions in teaching and non-teaching hospitals. Lancet 1957 Oct 19;273(6999):785-90.
17. Lee JA, Morrison SL, Morris JN. Case-fatality in teaching and non-teaching hospitals. Lancet 1960 Jan 16;1:170-1.
18. Hamburger S, Soffer A. Treating diabetic ketoacidosis: university center vs. community facility. J Am Med Womens Assoc 1982 Feb;37(2):52-4.
19. Lave JR, Fine MJ, Sankey SS, Hanusa BH, Weissfeld LA, Kapoor WN. Hospitalized pneumonia. Outcomes, treatment patterns, and costs in urban and rural areas. J Gen Intern Med 1996 Jul;11(7):415-21.
20. Cox JL, Chen E, Naylor CD. Revascularization after acute myocardial infarction: impact of hospital teaching status and on-site invasive facilities. J Gen Intern Med 1994 Dec;9(12):674-8.
21. Horsley BL, Cox WD, Schmitt EH, Sharp EH. Is coronary artery bypass in the community hospital justifiable? Am Surg 1977 Jun;43(6):372-6.
22. Bonett A, Roder D, Esterman A. Case-survival rates for infiltrating ductal carcinomas by category of hospital at diagnosis in South Australia. Med J Aust 1991 May 20;154(10):695-7.
23. Alter DA, Naylor CD, Austin PC, Chan BT, Tu JV. Geography and service supply do not explain socioeconomic gradients in angiography use after acute myocardial infarction. CMAJ 2003 Feb 4;168(3):261-4.
24. Ghali WA, Quan H, Brant R. Coronary artery bypass grafting in Canada: hospital mortality rates, 1992-1995. CMAJ 1998 Oct 20;159(8):926-30.
25. Mathoulin-Pelissier S, Salmi LR, Verret C, Demoures B. Blood transfusion in a random sample of hospitals in France. Transfusion 2000 Sep;40(9):1140-6.
26. Venturini F, Romero M, Tognoni G. Patterns of practice for acute myocardial infarction in a population from ten countries. Eur J Clin Pharmacol 1999 Jan;54(11):877-86.
27. Knaus WA, Draper EA, Wagner DP, Zimmerman JE. An evaluation of outcome from intensive care in major medical centers. Ann Intern Med 1986 Mar;104(3):410-8.
28. Wyatt SM, Moy E, Levin RJ, Lawton KB, Witter DM Jr, Valente E Jr, Lala R, Griner PF. Patients transferred to academic medical centers and other hospitals: characteristics, resource use, and outcomes. Acad Med 1997 Oct;72(10):921-30.
29. Taxis K, Barber N. Ethnographic study of incidence and severity of intravenous drug errors. BMJ 2003 Mar 29;326(7391):684.
30. Poses RM, Berlin JA, Noveck H, Lawrence VA, Huber EC, O'Hara DA, Spence RK, Duff A, Strom BL, Carson JL. How you look determines what you find: severity of illness and variation in blood transfusion for hip fracture.
    Am J Med 1998 Sep;105(3):198-206.
31. Petrik DW, McCready DR, Sawka CA, Goel V. Association between extent of axillary lymph node dissection and patient, tumor, surgeon, and hospital factors in patients with early breast cancer. J Surg Oncol 2003 Feb;82(2):84-90.
32. Meurer JR, Kuhn EM, George V, Yauck JS, Layde PM. Charges for childhood asthma by hospital characteristics. Pediatrics 1998 Dec;102(6):E70.
33. McCormick D, Fine MJ, Coley CM, Marrie TJ, Lave JR, Obrosky DS, Kapoor WN, Singer DE. Variation in length of hospital stay in patients with community-acquired pneumonia: are shorter stays associated with worse medical outcomes? Am J Med 1999 Jul;107(1):5-12.
34. Keenan SP, Busche KD, Chen LM, Esmail R, Inman KJ, Sibbald WJ. Withdrawal and withholding of life support in the intensive care unit: a comparison of teaching and community hospitals. The Southwestern Ontario Critical Care Research Network. Crit Care Med 1998 Feb;26(2):245-51.
35. Kahn KL, Pearson ML, Harrison ER, Desmond KA, Rogers WH, Rubenstein LV, Brook RH, Keeler EB. Health care for black and poor hospitalized Medicare patients. JAMA 1994 Apr 20;271(15):1169-74.
36. Herbst CA Jr, Elliott L, Koruda M, Maxwell JG. Laparoscopic cholecystectomy: comparison of university and community experience. Surg Laparosc Endosc 1993 Apr;3(2):95-9.
37. Cooper GS, Chak A, Harper DL, Pine M, Rosenthal GE. Care of patients with upper gastrointestinal hemorrhage in academic medical centers: a community-based comparison. Gastroenterology 1996 Aug;111(2):385-90.
38. Chen J, Radford MJ, Wang Y, Marciniak TA, Krumholz HM. Do "America's Best Hospitals" perform better for acute myocardial infarction? N Engl J Med 1999 Jan 28;340(4):286-92.
39. Barnett MJ, Kaboli PJ, Sirio CA, Rosenthal GE. Day of the week of intensive care admission and patient outcomes: a multisite regional evaluation. Med Care 2002 Jun;40(6):530-9.
40. Ayanian JZ, Weissman JS, Chasan-Taber S, Epstein AM. Quality of care for two common illnesses in teaching and nonteaching hospitals. Health Aff (Millwood) 1998 Nov-Dec;17(6):194-205.
41. Lipworth L, Lee JA, Morris JN. Case-fatality in teaching and non-teaching hospitals 1956-59. Med Care 1963;1:71-6.
42. Goel V, Olivotto I, Hislop TG, Sawka C, Coldman A, Holowaty EJ. Patterns of initial management of node-negative breast cancer in two Canadian provinces. British Columbia/Ontario Working Group. CMAJ 1997 Jan 1;156(1):25-35.
