# Supplementary material for: Patient Outcomes with Teaching Versus Nonteaching Healthcare: A Systematic Review
Source: PLoS Med. 2006 Sep 12;3(9):e341. doi: 10.1371/journal.pmed.0030341 (PMC1564172; doi:10.1371/journal.pmed.0030341)
Supplement: Supplementary file 3 [file pmed.0030341.st002.doc]

Supplementary information 2: Eligible studies having only unadjusted or non-usable data on mortality

| **Only non-adjusted data** | **Adjusted, but non-usable data** |
| --- | --- |
| Ashley (1971) | Agabiti (2003) |
| Bach (1998) | Aiken (1994) |
| Bottsford (1997) | Alter (2001) |
| Carpenter (1972) | Alter (2003) |
| Cole (1995) | Barry (2003) |
| Cooper (1996) | Chen (1999) |
| Elhanan (1995) | Flood (1984) |
| Feagan (2000) | Keeler (1992) |
| Fox (2002) | Lee-Feldstein (1994) |
| Gilchrist (1999) | Sloan (2000) |
| Goldacre (1976) | Teres (1983) |
| Gorst (1992) | Wolfe (1997) |
| Howard (2004) | Yuan (2000) |
| Huber (2001) | Peterson (1994) |
| Kingston (1992) | Kazmers (1996) |
| Mazer (1993) | Akkersdijk (1994) |
| Mylotte (2001) | Gregory (2001) |
| Shah (1971) |  |
| Sheng (2005) |  |
| Singh (1996) |  |
| Stiller (1994) |  |
| Stiller (1999) |  |
| Stone (1992) |  |
| Tanisada (2000) |  |
| Udvarhelyi (1990) |  |
| Wade (1994) |  |
| Wade (1996) |  |
| Whitehouse (2002) |  |
